# Best practice for wildlife gut microbiome research: A comprehensive review of methodology for 16S rRNA gene investigations

**DOI:** 10.3389/fmicb.2023.1092216

**Published:** 2023-02-22

**Authors:** Leigh Combrink, Ian R. Humphreys, Quinn Washburn, Holly K. Arnold, Keaton Stagaman, Kristin D. Kasschau, Anna E. Jolles, Brianna R. Beechler, Thomas J. Sharpton

**Affiliations:** ^1^Department of Microbiology, Oregon State University, Corvallis, OR, United States; ^2^Department of Biomedical Sciences, Carlson College of Veterinary Medicine, Oregon State University, Corvallis, OR, United States; ^3^School of Natural Resources and the Environment, University of Arizona, Tucson, AZ, United States; ^4^Department of Integrative Biology, Oregon State University, Corvallis, OR, United States; ^5^Department of Statistics, Oregon State University, Corvallis, OR, United States

**Keywords:** microbiome, 16S rRNA gene, ecology, wildlife, methodology, review

## Abstract

Extensive research in well-studied animal models underscores the importance of commensal gastrointestinal (gut) microbes to animal physiology. Gut microbes have been shown to impact dietary digestion, mediate infection, and even modify behavior and cognition. Given the large physiological and pathophysiological contribution microbes provide their host, it is reasonable to assume that the vertebrate gut microbiome may also impact the fitness, health and ecology of wildlife. In accordance with this expectation, an increasing number of investigations have considered the role of the gut microbiome in wildlife ecology, health, and conservation. To help promote the development of this nascent field, we need to dissolve the technical barriers prohibitive to performing wildlife microbiome research. The present review discusses the 16S rRNA gene microbiome research landscape, clarifying best practices in microbiome data generation and analysis, with particular emphasis on unique situations that arise during wildlife investigations. Special consideration is given to topics relevant for microbiome wildlife research from sample collection to molecular techniques for data generation, to data analysis strategies. Our hope is that this article not only calls for greater integration of microbiome analyses into wildlife ecology and health studies but provides researchers with the technical framework needed to successfully conduct such investigations.

## 1. Introduction

The advent of high-throughput DNA sequencing technologies has facilitated transformations in our understanding of the microbial biosphere. Until recently, the majority of microbial diversity was unseen, unidentified, and unstudied. Our newfound ability to interrogate the genomic information of microbes *in situ* has unlocked new understanding about the vast diversity of the microbial biosphere, the ecological distribution of microbes, and their linkage to key ecosystem services. One of the most rapidly accelerating areas of understanding that high throughput sequencing has unlocked is that of the integral role that microbes play in vertebrate (patho)physiological mechanisms. While a relatively small number of infectious, easily culturable microbes have been intensively studied, environmental DNA sequencing has revealed an extensive diversity of uncultured host associated microorganisms and has begun to uncover the complex interaction of commensals, mutualists, pathobionts, and pathogens that live in association with their vertebrate host (See Box 1 for definitions).

**BOX 1:** Definitions.

α-diversity	A measure of the diversity of the microbial community within a sample
Amplicon	A piece of DNA or RNA that can be the source or the product of a natural or artificial replication or amplification event, such as Polymerase Chain Reaction (PCR)
β-diversity	A measure of the similarity in terms of sample features (microbial composition) between pairs of samples
Chimeras	Amplicons that form from two different biological sequences, often occurring as a result of misreading a sample
Clade	A group of organisms believed to all have descended from a common ancestor
Commensals	Organisms in a relationship where one benefits, while the other is unaffected
Contigs	Shorter reads assembled into longer sequences based on matching overlapping regions
Degenerate primers	Primers in which a few bases are altered so that the primer will cover all the possible nucleotide combinations in the target protein; useful for amplifying the same gene (phylogenetic marker gene) from different organisms
Ecophylogenetics	A fusion of ecology with evolutionary history to determine how monophyletic lineages distribute with respect to ecologic metadata parameters of interest.
Mate pairs	Two fragments distal to each other in a genome and opposite in orientation that are produced during library preparation
Microbiome	The community of microorganisms (such as bacteria, fungi and viruses) and their genes, that inhabit a particular environment
Microbiota	The microorganisms that usually inhabit an environment, such as a plant or the human body
Mock communities	Sets of cells, genomes, or amplicons with known ratios that are used as controls to account for stochastic variation in microbiome studies
Monophyletic lineage	Descended from a common ancestor
Mutualists	Organisms in a relationship where both organisms benefit
Pathobionts	Organisms that can cause harm or promote pathology under certain genetic and/or environmental conditions
Pathogens	An agent that can cause disease, which could be a bacterium, virus or fungus.
PCR	Polymerase Chain Reaction (PCR) is a technique used in molecular biology to rapidly make up to a billion copies of a DNA gene target within a sample

In particular, the community of microorganisms that occupies the gastrointestinal tract, and their genes, collectively referred to as the gut microbiome, can play a central role in myriad aspects of vertebrate biology, including: digestion (Hanning and Diaz-Sanchez, 2015; Miller et al., 2020), metabolism (Koropatkin et al., 2012), growth (Yan et al., 2016), immune modulation (Thaiss et al., 2016; Levy et al., 2017; Sylvia and Demas, 2018) and pathogen defense (Khosravi and Mazmanian, 2013). In addition, gut microbiomes have been associated with neurological development (Lu et al., 2018) and behavior (Ezenwa et al., 2012; Archie and Tung, 2015; Lu et al., 2018), including mate selection (Sharon et al., 2010; Najarro et al., 2015; Rosenberg et al., 2018), thereby influencing selective advantages, such as mating success (Brucker and Bordenstein, 2013; Hird, 2017). The gut microbiome comprises a diverse set of microbial taxa, including bacteria, archaea, microbial eukaryotes and viruses, the composition of which can affect host physiology, where even low abundant taxa may be disproportionately impactful to their host. Diverse factors have been found to influence the gut microbiota community composition including diet, stress, and exposure to pollutants. Severely altered microbial community composition, or dysbiosis, has the potential to influence normal vertebrate homeostatic mechanisms, thereby manifesting patterns of microbial imbalance with clinical signs of disease. Various diseases have been found to associate with dysbiosis including increased susceptibility to infectious diseases (Bandera et al., 2018), malnutrition (Kumar et al., 2018), autoimmune diseases (de Oliveira et al., 2017; Wei et al., 2020), cardiometabolic disorders (Morel et al., 2020), and behavioral or cognitive impairments (Fröhlich et al., 2016; Noble et al., 2017; Sylvia and Demas, 2018). The relationship between the gut microbiome and host physiology is bidirectional; alterations in host physiology can affect the composition of the gut microbiome such as in the case of increased intestinal inflammation, which can differentially impede the growth of gut microbiota (Kamada et al., 2013; Halfvarson et al., 2017; Spiga and Winter, 2019) and vice versa.

The intimate association between the gut microbiome and host physiology has motivated recent efforts to consider the gut microbiome in the context of wildlife health, conservation, and management. Indeed, high-throughput DNA sequencing has already identified potential pathogenic bacteria in wildlife, thereby increasing our ability to monitor and mitigate zoonotic disease outbreaks (Galan et al., 2016). Understanding the possible influence of host-associated taxa on the evolution of a species is increasingly important to studies on wild vertebrates, particularly where insights could result in management protocols and extinction mitigation strategies for threatened species (West et al., 2019). However, our current knowledge of microbiomes is mostly limited to studies based on humans and model animal systems (Hird, 2017), with the majority of research focusing on the human gut microbiome (Davenport et al., 2017). This fact is problematic from a conservation standpoint because results from model animal systems are not necessarily representative of wildlife systems. A number of studies have shown the influence of captivity on the vertebrate gut microbiome (McKenzie et al., 2017) including fish (Dhanasiri et al., 2011), lizards(Kohl et al., 2017), parrots (Xenoulis et al., 2010), Antarctic seals (Nelson et al., 2013), chimpanzees (Uenishi et al., 2007), grizzly bears (Schwab et al., 2009), Tasmanian devils (Cheng et al., 2015), European wild rabbits (Funosas et al., 2021) and Namibian cheetah (Wasimuddin Menke et al., 2017). These differences between captive and wild gut microbiomes could have implications for wildlife management strategies such as captive breeding and species reintroduction programs. As a result, a growing number of studies have sought to characterize and evaluate the gut microbiome of wildlife populations (Couch et al., 2020, 2021; Sabey et al., 2020) with the objective of determining if the microbiome can serve as a useful resource for monitoring and managing the health of wild populations.

Owing to the often elusive, potentially dangerous nature or threatened/protected status of many wildlife species, the ability to obtain sufficient samples to make a meaningful contribution to the field can be a major limiting factor and constraint in many wildlife microbiome investigations. As such, wildlife microbiome investigations often coincide with samples being collected as part of veterinary inspections (Menke et al., 2015), or *ad hoc* collections from rehabilitated species [see DeCandia et al., 2019 for a skin microbiome investigation in three canid species]. The elusiveness of many wildlife species adds further complexity in that the exact time of fecal sample deposition is unknown, and exposure can lead to changes in microbial communities present in the sample (Menke et al., 2015). One potential caveat to account for this could be to conduct a small study of the target population, leaving fecal samples of known age exposed to the surrounding environment and sampling them at regular intervals to assess changes in microbiome changes over time. Menke et al. (2015) showed in two ungulate species in Namibia (giraffe and springbok) that microbiome composition changed little with environmental exposure over time, except for periods of moisture or light drizzle. In their case, the intermittent rain showers in a sense reactivated the microbial growth which had seemingly ceased owing to the hot desert conditions (Menke et al., 2015).

Another shortcoming of many wildlife microbiome investigations to date is the lack of repeated measures and longitudinal project designs. Primarily, this would be due to the logistics and costs involved in not only capturing and tagging or observing specific individuals within a population, but also the long-term investment required to resample the same individual over time (including telemetry equipment and personnel time). Moreover, if deposition of the fecal sample is not witnessed, genotyping may be necessary to confirm that the collected sample belongs to the target animal. These may be additional project and personnel costs that should be incorporated into wildlife microbiome investigation designs. Despite this, the inclusion of longitudinal time-series data tracking changes in gut microbiome composition will allow researchers to address questions such as whether the gut microbiomes of individuals within a population will respond synchronously or asynchronously to shifting environmental resources (Björk et al., 2022). Human research suggests that the gut microbiome can change rapidly in response to environmental change, often with individual health and fitness consequences (Björk et al., 2019). As such, longitudinal studies detecting differences in synchronicity of gut microbiome response to changing environmental resources could elucidate shared microbiota-associated traits, such as differences in susceptibility to disease (Björk et al., 2022). Furthermore, longitudinal studies will allow researchers to determine the impact that host population structure and sociality has on individual gut microbiome composition (Murillo et al., 2022).

The progress and inferences made from many human microbiome studies, owe their success largely to efforts such as the Human Microbiome Project (HMP) dedicated to characterizing the human microbiome at 5 different body sites (Turnbaugh et al., 2007). Consequently, many of the gut bacteria for humans have been sequenced and classified taxonomically, with sequences stored in searchable databases (Turnbaugh et al., 2007; Hamady and Knight, 2009; Wylie et al., 2012). The same is not necessarily true for wildlife (Couch and Epps, 2022). For some species, this problem may not be as drastic as often wildlife species will have a well-studied domesticated counterpart [e.g., with ruminants such as domestic cattle vs. African buffalo (Couch et al., 2021) or domestic sheep vs. Desert bighorn sheep (Couch et al., 2020)] where many gut bacterial taxa may be shared, allowing for greater precision when taxonomically annotating 16S rRNA gene sequences from these host species. While this lack of referential taxonomic classification for less-studied wildlife species may challenge initial investigations (Couch and Epps, 2022), this limitation could also be viewed as a timely opportunity to describe and characterize the taxonomic diversity of these less well studied systems.

Efforts to characterize the gut microbiome involve a variety of techniques that must be accurately implemented to ensure meaningful outcomes. Perhaps the most common approach used to classify microbial taxa is the sequencing of universally conserved, taxonomically diagnostic phylogenetic marker genes, the most characterized of such genes being the small subunit ribosomal RNA (16S rRNA) gene or 16S rDNA. By sequencing the 16S rRNA genes of the various taxa that comprise an archaeal and bacterial microbial community with high-throughput sequencing technology, researchers can quickly and inexpensively determine which organisms comprise the community, quantify biodiversity, and measure the phylogenetic relatedness of these organisms.

While this approach is powerful, it requires the implementation of several key steps prior to bioinformatic analysis. First, biological specimens, such as environmental or host-associated samples (e.g., feces), that contain microorganisms need to be collected and preserved in ways that avoid contamination or bias. The DNA from the organisms that comprise each sample is then simultaneously extracted to enable DNA-based inferences of community composition. As a result, DNA extraction techniques that are biased in the efficiency with which the cells are lysed can yield biased interpretations of community composition. Moreover, degenerate primers are often used to amplify through PCR a specific genomic locus (e.g., hypervariable regions of the 16S rRNA gene) from each of the genomes present in the sample. Here, biases can result from the selection of primer or PCR conditions. Finally, after the PCR amplicons are sequenced, a variety of bioinformatic approaches can be used to analyze the sequences and test hypotheses, but different approaches may reveal different patterns in the resulting data.

**Figure 1:**
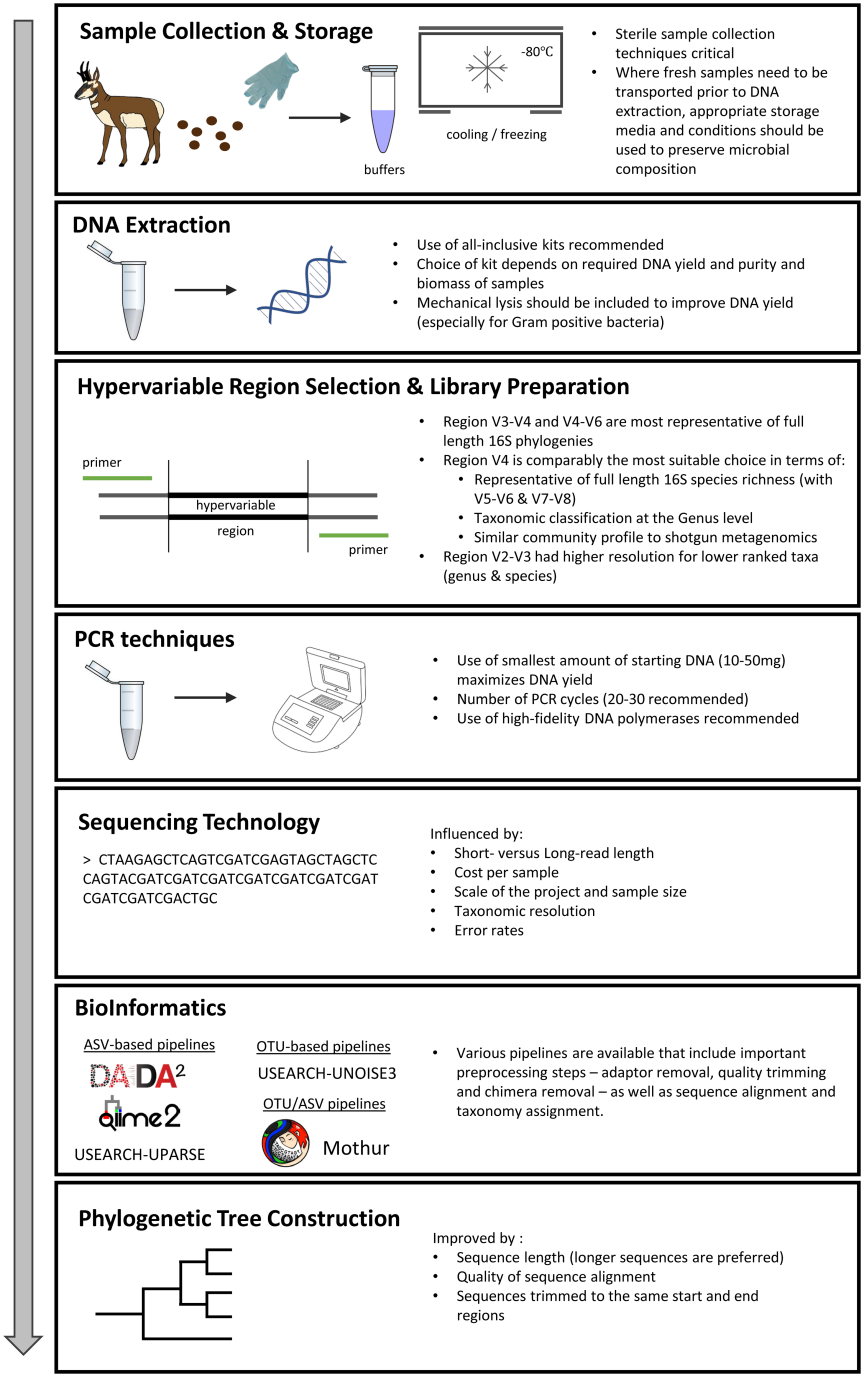
Points of consideration in 16S rRNA gene analyses of wildlife gut microbiome investigations.

The specific analytical approaches used can, in some cases, dramatically impact conclusions. Therefore, to maximize the impact of the addition of microbiome research to wildlife population studies and to assist researchers wishing to embark on a microbiome-based investigation, we have reviewed and summarized the state of knowledge on various study parameters, including: sample storage and preservation techniques, PCR, mock communities and batch effects, hypervariable region selection, sequencing platforms, and bioinformatic pipelines, and how different decisions at each stage can affect inferences about the bacterial communities of interest. While prior reviews have clarified best practices in a more general sense (see Knight et al. (2018) and Quince et al. (2017)), or have focused on domestic livestock (Weinroth et al., 2022) and companion animals (Jarett et al., 2021), we focus our discussion specifically around points of consideration for incorporating 16S rRNA gene analyses into wildlife investigations (See Figure 1 and Supplementary Decision Tree Flowchart).

## 2. Sample collection and preservation techniques

After designing well controlled and powered studies, microbiome investigations begin by collecting biological specimens. In the case of gut microbiome investigations, this typically involves obtaining a fecal sample from the individual hosts being studied in part because stool samples provide non-invasive access to the gut microbiome. In some systems, tissue biopsies (e.g., in tranquilized animals) or collection of lumenal contents of specific regions of the gastrointestinal tract (e.g., in fistulated or necropsied animals) are possible. Regardless, all samples must be collected using sterile techniques to avoid contamination by the researcher. Additionally, samples need to be preserved and stored to mitigate subsequent microbial growth. Here, we discuss points of consideration regarding gut microbiome sample collection and preservation (summarized in Supplementary Table 1).

Many ecological studies require the use of environmental and host-associated samples containing microbial biomass to be collected long before DNA extraction. For non-invasively obtained fecal samples, collection should be as soon as possible after defecation (Amato et al., 2013). Changes in sample microbial composition, particularly the ratio of anaerobic to facultative aerobic and aerobic bacteria have been shown to occur with increased time of exposure of samples to the external environment (Menke et al., 2015). Care should also be taken during collection and processing, to exclude those parts of a sample that may have been contaminated by the ground or surrounding environment (Amato et al., 2013). An array of preservation techniques has been developed to stabilize microbial DNA to ensure more accurate detection of microbial taxa at a later date. Several studies have assessed the impact of preservation on the accuracy of estimates of patterns in microbiome variation, including sample and community composition [α- and β-diversity (See Supplementary Table 1)]. α-diversity measures the diversity of the microbial community within a sample, whereas β-diversity is a measure of the similarity in terms of sample features (microbial composition) between pairs of samples (Knight et al., 2018). Some studies have shown little effect of preservation method on α-diversity measures (Chen et al., 2019; Moossavi et al., 2019) and several studies attribute the largest difference in microbial community composition to inter-sample or inter-subject variation (Carruthers et al., 2019; Chen et al., 2019; Moossavi et al., 2019; Lim et al., 2020). However, choice of storage method may affect frequencies of bacteria more than their presence/absence (Song et al., 2016).

Microbiome preservation methods can be grouped into three main categories, being cold storage, buffer solutions and dry storage / card preservation. It is important to note that each preservation and storage method produces unique inherent biases in 16S rRNA gene-based studies and as such no perfect procedure exists. Storing samples in temperature-controlled environments can reduce variation in microbial communities that can occur over time. Generally, −80°C storage of biological and environmental samples or cryopreservation is regarded as the highest fidelity storage temperature or “gold standard” to preserve DNA quality and ensure accurate microbial community profiles (Tzeneva et al., 2009; Lauber et al., 2010; Bahl et al., 2012; Choo et al., 2015; Fouhy et al., 2015; Vandeputte et al., 2017; Carruthers et al., 2019; Chen et al., 2019; Moossavi et al., 2019; Marotz et al., 2021). Applying cryopreservation techniques in a field situation could be difficult, although it may be feasible if one can obtain liquid nitrogen or dry ice at or near one’s field site.

Storage temperature has been shown to affect abundance-weighted β-diversity (Song et al., 2016). Relative abundance estimates have also been shown to vary by sample storage temperature ranging from −80°C to approximately 25°C (Roesch et al., 2009; Lauber et al., 2010; Bahl et al., 2012; Choo et al., 2015; Gorzelak et al., 2015). Some gut microbiome studies in humans have reported significant shifts in the abundance of the phyla Firmicutes and Bacteroidetes between samples stored at different temperatures (Bahl et al., 2012; Gorzelak et al., 2015). Variation in the ratios of these phyla may obscure biologically meaningful results because the ratio of Bacteriodetes to Firmicutes in fecal samples is often evaluated as an indicator of host health (Ley et al., 2005; Koliada et al., 2017). Conversely, other studies have found that there are no significant differences between the relative abundance of major phyla in gut microbiome samples stored in differing temperatures without buffers or subjected to two thaw cycles (Dominianni et al., 2014; Bassis et al., 2017).

The effects of storage temperature on microbial communities may also be biome specific. Although minimal variation in microbial community composition has been associated with storage temperatures for human oral (Luo et al., 2016), skin (Lauber et al., 2010) and vaginal microbiome samples (Bai et al., 2012) stored in buffer solutions, the converse is true for free-living soil communities. The community composition of soil samples stored at room temperature for up to 14 days was mostly unaffected (Lauber et al., 2010); however, air-dried soil samples stored for 3 months exhibited significant differences in richness and diversity of bacterial profiles compared to samples stored at −80°C (Tzeneva et al., 2009).

Some preservation solutions (*OMNIgene.GUT* buffer and *Whatman* FTA cards) were shown to result in lower compositional changes in freshly sampled fecal samples compared to others (*RNAlater*, 70% ethanol and 95% ethanol), however *Whatman* FTA cards consistently produced higher diversity values (Song et al., 2016). Another study showed that samples preserved in OMNIgene.GUT were more similar to cold-stored samples, generally considered to stabilize DNA, than replicates stored in RNAlater, Tris-EDTA, or at room-temperature (Choo et al., 2015). When cooling is unavailable, card-based preservation methods such as fecal occult blood test (FOBT) or Whatman FTA cards may be better choices than buffer solutions (Dominianni et al., 2014; Sinha et al., 2016; Song et al., 2016). However, according to the manufacturer, OMNIgene.GUT can preserve microbial composition at ambient temperature for 60 days (Doukhanine et al., 2016). Recent studies showed that OMNIgene.GUT maintained microbiome profiles for 21 days (Lim et al., 2020) and preserved β-diversity weighted unifrac stability for 48 h at room temperature (Liang et al., 2020). In a study on rats, Ma et al. (2020) found MGIEasy to be superior for DNA concentration than OMNIgene.GUT and LongSee at ambient temperature. Another study showed NBgene.GUT to be as effective as OMNIgene.GUT at preserving the relative abundance of dominant and functional bacteria in human stool samples compared to frozen controls (Park et al., 2020). Similarly, Chen et al. (2019) showed Norgen Biotek to be comparable to OMNIgene.GUT, CURNA, DNA Genotek HEMA and RNAlater buffer solutions in maintaining β-diversity microbial composition.

Use of RNAlater, however, may result in decreased DNA purity and lower microbial diversity (Dominianni et al., 2014), higher variation in microbial communities with heat (Song et al., 2016), and reduced DNA yields (Gorzelak et al., 2015). Preservation in 70% ethanol was found least effective at stabilizing community structure and yielded similar results to using no preservative measures (Song et al., 2016). Consequently, if ethanol preservation is used, concentrations of at least 95% should be used to reduce preservation biases, particularly where freezing is unavailable and for prolonged sample storage in ambient or sub-optimal conditions (Hale et al., 2015; Song et al., 2016).

Additional considerations when selecting between available preservation techniques include potential for conducting further analyses and whether the study is longitudinal in nature. For example, samples stored in RNAlater can be used for downstream transcriptomic investigations and samples stored in ethanol can be used for metabolomics studies (Sinha et al., 2016). Should multiple molecules need to be extracted from a single sample, preservation using a fixative suitable to various types of molecules (such as 95% ethanol) may be preferable (Song et al., 2016). Marotz et al. (2021) showed 95% ethanol to be an effective storage preservation method for several weeks at room temperature. For studies following individuals or populations over time with repeated sampling to measure changes in microbiome communities, it is imperative that the same sampling protocol and sample storage preservation methods be employed to avoid confounding differences in community composition with sample preservation techniques. Therefore, the sample preservation method should consider planned analyses, future sample collections from the same individuals or populations, and potential future uses of samples to investigate the biological question(s) of interest.

In summary, based on the findings of our literature search (See Supplementary Table 1), when samples cannot be processed shortly after collection, storage of microbial samples using OMNIgene.GUT buffer solution, >95% ethanol, cryopreservation or freezing at a maximum temperature of −80°C yields the most stability in microbial community composition. New preservation methods that enter the market may yield similar or improved results. Due to the diversity of DNA preservation methods employed in conjunction with temperature storage, it is difficult to disentangle absolute guidelines. Further work should be conducted to elucidate the effects of sample preservation and long-term storage strategy on the integrity of microbial community DNA across different microbiomes. However regardless of methodology, we stress the importance of preservation consistency across samples to reduce batch effects.

## 3. DNA extraction

Once samples have been preserved and stored, the next goal is extraction of the greatest yield and purest quality of DNA possible. Choice of DNA extraction method can influence both the concentration and quality of the DNA obtained from the assay (Nechvatal et al., 2008). Here, we discuss the effects of different DNA extraction methods (e.g., enzymatic vs. mechanical cell lysis) on DNA purity and yield (summarized in Supplementary Table 2).

In the age of high-throughput sequencing, biotech companies have engineered all-inclusive kits to expedite extractions and standardize methodology. Depending on the extraction method, researchers have reported varying yields of DNA (Nechvatal et al., 2008) and purity of nucleic acids (Gerasimidis et al., 2016; Szopinska et al., 2018). DNA yield and purity have been shown to result in differing community diversity and abundance estimates. Yet despite improvements, and regardless of method, biases are introduced during DNA extraction (Yuan et al., 2012; Brooks et al., 2015) and must be considered in study design.

Fecal microbiome samples will reasonably contain a certain amount of undigested raw food remains, which will differ based on dietary preferences, and which may be of particular concern to wildlife microbiome investigations. Chloroplast and mitochondrial sequences included in the extraction could result in off-target amplification and could impact the resulting microbiome profile. In certain cases where it is vitally important for researchers to determine the relative contributions of diet to the diversity and composition of the fecal microbiome compared to other factors under study, similar methods that are utilized to identify microbial taxa can be used to identify dietary constituents. For herbivorous animals, the internal transcribed spacers of the nuclear ribosomal loci can be used to identify various plant species in a given fecal sample (Iwanowicz et al., 2016), for carnivorous animals, sequencing the partial mitochondrial control region can identify mammalian prey species (Shi et al., 2021), and for insectivorous animals, sequencing mitochondrial cytochrome oxidase subunit1 can similarly identify insect prey species (Esnaola et al., 2018). For carnivores insectivores, these methods could be coupled with a variety of direct examination methods for determining diet from fecal samples (Klare et al., 2011).

An additional aspect for consideration is that depending on the physical properties of the microorganisms present in the sample, DNA extractions that only incorporate standard chemical lysis may be unable to access DNA from the whole microbial community. Organisms such as *Mycobacterium* spp. and *Bacillus* can form spores which contain thick cell walls that require mechanical lysis techniques to recover DNA (Kuske et al., 1998; Vandeventer et al., 2011). Mechanical lysis *via* bead beating has been shown to reduce biases during DNA extraction that affect downstream community calculations of richness and relative abundance estimations due to the inability to access DNA from subsets of bacterial and archaeal populations (Kuske et al., 1998; Carrigg et al., 2007; de Boer et al., 2010; Salonen et al., 2010; Smith et al., 2011; Yuan et al., 2012). Furthermore, while there are a multitude of different options for mechanical lysis, 0.1 mm silica beads have improved the recovery of Gram positive bacteria during DNA extractions without negatively impacting Gram negative organisms (de Boer et al., 2010).

Choice of DNA extraction method affects the overall DNA concentration obtained from samples, although conflicting evidence exists as to which method and kit recovers the most accurate and highest quality DNA. In human fecal samples, use of the QIAamp DNA Stool Kit (QIAGEN) for DNA extractions was shown to produce higher average DNA yields than extractions using the MoBio Fecal Kit (now owned by QIAGEN) (Nechvatal et al., 2008). Similarly, Szopinska et al. (2018) found that the QIAamp DNA Stool Mini Kit yielded greater DNA concentrations and higher DNA purity compared to the MoBio PowerFecal DNA Kit (now owned by QIAGEN). Additionally, use of the QIAamp DNA Stool Mini Kit for extracting DNA produces better nucleic acid purity, greater sequencing yield, longer reads after quality trimming, and higher OTU-level diversity than phenol-chloroform or chaotropic salt based DNA extractions, yet lower double stranded DNA yield than chaotropic salt DNA extractions (Gerasimidis et al., 2016).

Based on the above studies, the QIAamp DNA Stool and Stool Mini Kits would be obvious choices for DNA extraction kits (See Supplementary Table 2). However, Bahl et al. (2012) found that the PowerSoil DNA Isolation Kit (now owned by QIAGEN) resulted in higher DNA yield than the QIAamp DNA Stool Kit. Furthermore, Panek et al. (2018) found that the MP Biomedicals Fast DNA Spin kit for feces outperformed both the QIAamp DNA Stool Mini Kit and PowerSoil DNA Isolation Kit in terms of DNA yield and purity. A third study, comparing five commercial kits, highlighted the QIAsymphony Virus/Bacteria Midi Kit as producing the highest quality DNA, and along with the Zymo ZR Fecal DNA MiniPrep Kit, produced the highest DNA yields and bacterial diversity (Claassen et al., 2013). The PowerSoil DNA Isolation Kit, however, has been found to be more effective than the QIAamp DNA Stool Kit for low bacterial biomass samples (Velásquez-Mejía et al., 2018) and was the kit selected for research conducted by the Human Microbiome Project (Huttenhower et al., 2012) as well as the Earth Microbiome Project (Thompson et al., 2017; Caporaso et al., 2018).

Another consideration is that estimates of relative abundance for microbial taxa may be biased by DNA extraction method (Yuan et al., 2012; Wesolowska-Andersen et al., 2014; Brooks et al., 2015; Velásquez-Mejía et al., 2018). For example, use of the MoBio PowerSoil DNA Isolation Kit resulted in an increased number of Firmicutes and Actinobacteria and a decrease in Bacteroidetes compared to samples extracted using a QIAamp DNA Stool mini Kit (Velásquez-Mejía et al., 2018). The choice of extraction kit may also be influenced by the target microorganism(s). Menu et al. (2018) showed that the EZ1 (Qiagen) kit yielded higher concentrations on nucleic acids and lower levels of contaminants than the QIAamp DNA Stool Mini Kit, for pathogenic eukaryotes (5 protozoa and 1 microsporidium).

One drawback for all sequence-based assessments of microbial communities is that the data are inherently compositional and very often highly sparse, which can lead to spurious correlations between taxon abundances and metrics of interest or between taxon abundances themselves, when using traditional statistical methods. Others have provided an overview of the various tools for dealing with both the compositional and sparse nature of microbiome data (Tsilimigras and Fodor, 2016; Gloor et al., 2017). Beyond statistical tools, researchers may want to quantify absolute counts of bacterial cells/genomes to properly scale counts from sequencing. Again, there are a number of options for researchers including various microscopy, flow cytometry, and PCR-based methods for targeting all cells, only live cells (at the time of sample collection), or even specific taxa that may be of relevance to the study system (Jespers et al., 2012; Wang et al., 2021).

In summary, we recommend mechanical lysis if this is not already integrated into the kit protocol to maximize microbial diversity recovered from samples and minimize taxa-specific biases during DNA extraction. It is difficult to identify a single optimal DNA extraction method, as some studies claim that the choice of kit significantly impacts the resulting microbial profiles (Maukonen et al., 2012), whereas others report that the ability to isolate bacteria was reproducible across all kits tested (Claassen et al., 2013; Greathouse et al., 2019). Additionally, we stress that a single method of DNA extraction should be executed for all samples within a given study to negate inter-sample biases.

## 4. Mock communities

Sources of error and bias can occur at any stage of the microbiome investigation, including technical variation inadvertently introduced by the researcher. One way to assess this bias is to include mock microbial communities in the research design. Mock communities are a defined set of cells, genomes, or amplicons with known ratios that are used as controls to account for stochastic variations that occur during the various preparatory steps of microbiome studies (Yeh et al., 2018). Additionally, mock communities used at the onset of a study can help detect primer bias toward important and abundant clades (Parada et al., 2016). Mock communities help to ensure that “normal” sequencing occurs and not “aberrant,” meaning that sequences may be up to two-fold greater or lower than they would be in a “normal” run indicating a loss of precision. In these “aberrant” runs taxa abundance may be greatly misrepresented and sequences of rarer taxa may be lost completely (Yeh et al., 2018). When selecting a mock community to use, the researcher is given three choices for mock community type and can create their own or purchase a commercially available one.

Mock communities may be: 16S sequence copies, genomic, or whole cell. 16S and genomic communities can be used somewhat interchangeably provided the researcher is cognizant that the number of 16S copies per genome is variable between taxa, even between those that are closely related (Stoddard et al., 2015). On the other hand, a researcher may elect to use whole cell mock communities to additionally control for DNA extraction variability. Mock communities should be included in analyses along with other samples. For genetic material, mock communities can be included from the PCR step, whereas whole cell mock communities should be used from the DNA extraction step. Once the decision between genetic material versus whole cell mock communities has been made, there are then two options to source the mock community. Mock communities can be made *de novo* by the researcher or a pre-made mock community may be purchased from several suppliers including American Type Culture Collection (ATCC) and Zymo Research. When choosing which mock community to use, the most important consideration is that it contains the clades with characteristics of interest for the purposes of the study (i.e., gram-positives, gram-negatives, gammaproteobacteria, fungi, archaea, GC rich sequences, etc.). Preliminary data on the microbiome in question may be needed to determine which taxa should be included in the mock community. Additionally, mock communities can be selected or constructed to contain genetically distinct 16S rRNA genes which can be filtered out during data analysis to prevent contamination of samples with sequences from the mock community. We recommend using mock communities that contain sequences related to the most abundant, significant, and ubiquitous organisms in the microbiome community of interest, as well as any organisms that are of interest to the researcher, such as low abundance but omnipresent organisms.

## 5. Batch effects

Bias resulting from technical variation introduced by the researcher is practically unavoidable in microbiome studies. Such unwanted variation will be referred to here as “batch effects.” In an ideal situation, researchers would account for possible effects with experimental and protocol design from the start of a study. Where possible, biological variation of interest should not be conflated with sampling regimes, differences in protocol, or, when dealing with large numbers of samples, sub-setting of samples for processing. Researchers should try to ensure that factors such as age/sex/genetics of their samples, sampling location/time, kit type/processing time, etc. (Wang and Lê Cao, 2020) do not overlap to large degrees with the actual biological variation they are testing in their experiments. If, however, such conflation is unavoidable due to the nature of the study system, there are a number of *post hoc* statistical computational methods that have been developed for dealing with such batch effects, specifically for microbiome data (Gibbons et al., 2018; Ma et al., 2020; Wang and Lê Cao, 2020).

Regardless of the study system, batch effects from sample processing can and should be accounted for by all researchers, and minimized where possible (Chen et al., 2019). When sub-setting samples for processing, it is important to include roughly equal proportions of samples representing the biological variation of interest in each subsample and to randomize the samples across plates or racks of tubes. Processing samples in this way can not only reduce the general batch effects that might arise from accidental technical variations between subsets but can help minimize the impact of both well-to-well and background contamination, which are known problems with both plate and tube-based methods of microbiome sample processing (Minich et al., 2019).

## 6. Hypervariable region selection

In 16S rRNA gene studies, following DNA extraction, specific subregions of interest within the 16S rRNA gene need to be amplified using the polymerase chain reaction (PCR). Although high throughput DNA sequencing has allowed us to produce 10^7^–10^8^ sequences per run, the technical limitations of the most commonly applied sequencing platforms result in short length reads (100–400 bp; Schloss, 2010) of the 16S gene, which is approximately 1,500 bp in length. Within this gene there are nine so-called hypervariable regions (V1-V9) that manifest relatively higher mutation rates, flanked by relatively conserved regions of DNA. These hypervariable regions are useful to sequence because they provide resolved insight into the divergence between relatively closely related microbial taxa, while the conserved sequences flanking these hypervariable regions make for useful PCR priming sites to amplify 16S genes of diverse taxa (Baker et al., 2003). However, an important question is often raised: which hypervariable region(s) should be targeted in a 16S rRNA gene sequence survey? In this section, we discuss how the selection of different hypervariable regions influence downstream microbiome analysis results (summarized in Supplementary Table 3). However, we note that the growing trend of long-read sequencing and shotgun metagenomics may mitigate the need to prioritize specific hypervariable regions in the near future (Sharpton, 2014).

Rates of nucleotide conservation and hypervariable region length vary, consequently dictating the efficacy of each region to differentiate between taxa. Researchers have extensively considered how the use of DNA sequences from the different hypervariable regions impact study outcomes, such as phylogeny-based measurements, taxonomic classification rates, and community diversity metrics. Phylogenies reconstructed using V4-V6 region sequences (Yang et al., 2016) and V3/V4 sequences (Ragan-Kelley et al., 2013) are most representative of full-length 16S phylogenies while V2, V8 (Yang et al., 2016) and V9 (Ragan-Kelley et al., 2013) were found least similar to the full-length phylogenies (see Supplementary Table 3). Bukin et al. (2019) found the V2-V3 region to be preferable to the V3-V4 region in terms of classification for lower ranked taxa (genus and species) using samples from an aquatic environment. However, across sampling environments, the V4 region sequences, on average, have been shown to be best at annotating sequences with genus level taxonomic labels (Soergel et al., 2012) and the most accurate when using simulation and mock community data (Liu et al., 2020).

β-diversity metrics applied to 16S data have been shown to be robust to primer and sequencing platform selection (Tremblay et al., 2015). Of those tested (V4, V6-V8, and V7-V8), the V4 hypervariable region sequences most closely resembled community profiles obtained using shotgun sequencing (sequencing of random DNA strands; Tremblay et al., 2015). Similarly, in another study, simulated V4, V5-V6, and V6-V7 hypervariable region fragments most closely estimated full-length 16S sequence species richness (Youssef et al., 2009).

In addition to the particular variable region of interest sequenced, the primer sequence itself can lead to biases during amplification. For example, the Earth Microbiome Project 16S Illumina Amplicon Protocol^1^ specifically modifies the V4 515F – 806R primer pair (Caporaso et al., 2011) to enable longer amplicon (e.g., the V4 V5 region using 515F-926R; Quince et al., 2011; Parada et al., 2016) as well as addition of degeneracy to forward and reverse primers to decrease bias against particular microbial lineages (Apprill et al., 2014; Parada et al., 2016). Use of the original primer pairs resulted in decreased detection ability of particular microbial lineages such as Crenarachaeota and Alphaproteobacterial clades (e.g., SAR11). Similarly, Chen et al. (2019) found an inability of the degenerate primer 27f-YM to detect the majority of Bifidobacteriales, and other studies have demonstrated how primer choice can influence relative abundance estimations (Tremblay et al., 2015; Liu et al., 2020).

## 7. Polymerase chain reaction based library preparation

In many microbiome studies (and metabarcoding in general), PCR serves a dual purpose: it amplifies a genomic locus of interest to ensure there is a sufficient amount of DNA to sequence and it prepares the DNA for sequencing on a DNA sequencing platform (i.e., library preparation). However, errors can be introduced during PCR that affect downstream analyses. These errors are often difficult to detect (Goodrich et al., 2014) and can be compounded with each additional amplification cycle. This section highlights techniques employed to reduce potential errors during PCR.

Potential PCR errors could arise from poor DNA polymerase fidelity, resulting in substitutions, insertions, and deletions of base pairs, as well as off-target primer binding. Consequently, these errors could potentially produce chimeras arising from incompletely extended sequences annealing to another sequence. Such errors can significantly impact estimation of microbial community diversity and composition.

Further sources of error potentially affecting the efficacy of the PCR reaction could result from the choice of PCR reagents, such as the specific Taq enzyme used, or could relate to the properties of the samples themselves, which will vary in the amount of PCR inhibitors present and carried through downstream DNA extraction reactions. One solution to this would be to fine-tune the amount of DNA utilized in the reaction, such as reducing the concentration of a DNA aliquot that contains large amounts of PCR inhibitors or increasing the concentration of DNA if initial reactions fail. To account for these complications, researchers can follow established reputable protocols, such as the Earth Microbiome Project 16S Illumina amplicon protocol (Caporaso et al., 2018), which includes the use of DNA extraction kits known to both effectively remove PCR inhibitors while applying seemingly robust reagents that reliably amplify the specified amount of DNA.

Minimizing the number of PCR cycles and using high fidelity DNA polymerases (such as KAPA) has been shown to help alleviate the formation of chimeras, nucleotide polymorphisms, and compositional biases in microbial communities (Gohl et al., 2016; Sze and Schloss, 2019). Using mock communities, Sze and Schloss (2019) demonstrated that the number of PCR cycles is of primary importance, with polymerase choice being secondary. When comparing the efficacy of various polymerases, after clustering sequences to reduce noise and with 30 cycles of PCR amplification, KAPA polymerase had the lowest error rate followed by Phusion, Q5, Accuprime, and Platinum, although Accuprime generated the fewest chimeras (Sze and Schloss, 2019). As additional cycles of PCR were conducted, Shannon diversity index generally increased and bacterial communities became more even (Sze and Schloss, 2019). For this reason, Sze and Schloss (2019) caution against comparing data created under differing PCR conditions. Another study found that beyond 20 cycles of PCR, KAPA polymerase outperformed Q5 and Taq both in having the lowest nucleotide error rate and least number of chimeric sequences (Gohl et al., 2016). Additionally, reducing the amount of starting material (to between 10 and 50 mg wet weight fecal samples) used in PCR increases DNA yield (Ariefdjohan et al., 2010), and decreases the percentage of chimeric reads detected after DNA sequencing (D’Amore et al., 2016). Sample biomass has also been shown to be the most important factor in determining representative microbial composition (Villette et al., 2021). We recommend using high fidelity DNA polymerases and minimizing the number of PCR cycles and amount of starting DNA used to mitigate any potential errors arising from the PCR process.

Previous best practice also suggested that, to minimize bias, it is advisable to conduct triplicate PCRs per sample (Goodrich et al., 2014). In a recent study spanning hundreds of samples from different environments, results from single PCR reactions were found similar to pooled results from triplicate runs (Marotz et al., 2019). This suggests that owing to the improved processivity and fidelity of DNA polymerases, the need for triplicate runs may be obsolete, substantially reducing costs (Marotz et al., 2019). The authors do add a caveat to this claim, however, stating that prior tests should be run for the specific sample environment prior to abandoning conventional wisdom (Marotz et al., 2019).

## 8. Sequencing technology

Following DNA extraction and amplification, the genes present in each sample need to be sequenced. Next generation sequencing (NGS) employs parallel sequencing technology and as the technology evolves, so too does the number of commercially available NGS platforms. In this section, we explore the current options available to researchers and the advantages and disadvantages associated with each.

DNA sequencing has evolved from the original 2D gel electrophoresis (1975), Sanger sequencing (1977), and more recently Roche 454 (2004–2012). Illumina’s (~2007) HiSeq and MiSeq sequencing platforms have quickly become the sequencing standard, producing a higher quantity and quality of reads than Roche 454 (Caporaso et al., 2012). The two Illumina sequencers (HiSeq and MiSeq) can be distinguished from each other by scale of operation, cost, and read length. MiSeq machines deliver rapid smaller scale sequencing while the HiSeq reduces the cost per sample by enabling higher parallelization at the expense of time and sequence length (Caporaso et al., 2012). MiSeq and HiSeq have both been shown to produce low variability across lanes in a single run and similar quality reads (Caporaso et al., 2012). Taking advantage of the higher quantity of reads, dual-index paired-end primers have enabled MiSeq reads to attain similar error rates to Roche 454 GS-FLX Titanium while increasing read-depth by 10-fold (Kozich et al., 2013). Unfortunately, MiSeq is currently limited to short read sequencing of roughly 300 nucleotides. Attempts to increase read length of MiSeq generally resulted in reduced overlap between read pairs (Schloss et al., 2016).

While Illumina’s HiSeq/MiSeq platforms limited researchers to short hypervariable regions of the 16S gene, emerging long-read sequencing technologies such as PacBio and Oxford Nanopore hold the potential to transform 16S investigations by offering access to full-length 16S gene sequence reads. When applied to 16S rRNA gene amplicon sequencing, these platforms resolve circular consensus sequences (CCS) and unique molecular identifiers (UMI; Callahan et al., 2019; Karst et al., 2021), which are relatively long 16S sequences than typically obtained by Illumina platforms. These longer read 16S sequences provide more information about the genomic composition of each sequenced molecule and are more likely to receive better resolved taxonomic annotations to the level of genus or species (Schloss et al., 2016; Pootakham et al., 2017) and produce phylogenies more similar to those reconstructed using full-length genes (Ragan-Kelley et al., 2013). One limitation of long-read sequencing technologies that has reduced their adoption is concern surrounding their higher sequencing error rates compared to short read technologies. Rapid improvements to these technologies, however, are leading to the development of new informatic solutions targeted at reducing long-read errors. For example, after conducting read filtering and quality control, PacBio (P6-C4 chemistry) can produce sequences with error rates of around 0.03% (Schloss et al., 2016; Wagner et al., 2016). Another potential effect of long-read sequencing is on the improved accuracy of estimates of species richness (Jeong et al., 2021). One study found that MiSeq V1-V2 sequences have elevated species richness estimates compared to PacBio full-length sequences from the same sample (Wagner et al., 2016). However, when the full-length PacBio sequences were truncated to simulate V1-V2 reads, there was an increase in species diversity indicating that short read sequencing may result in an overestimation of species diversity (Wagner et al., 2016). In addition, the use of full length sequences including all hypervariable regions, improved classification of the majority of sequences at the species level (Johnson et al., 2019; Jeong et al., 2021).

As new sequencing platforms are developed and chemistries improve, the per nucleotide error rates resulting from sequencing error will likely decrease. Currently, a large factor in platform selection resides in cost, wherein HiSeq is often the cheapest in per sample cost, followed by MiSeq and then PacBio. Unfortunately, read length and read quality are proportional to cost (Amir et al., 2017). Longer read platforms tend to sequence a smaller number of molecules from the community, and as a result tend to require higher costs to characterize the diversity of the overall community as compared to short read (but high volume) platforms. These long read approaches hold great potential for advancing 16S analyses, but our recommendation is to focus their application toward specific questions (e.g., phylogenetic inference of the abundant taxa across communities) unless comprehensive characterization of a community is not a critical priority. Therefore, the selection of a sequencing platform should be based on experimental need. The following sections which discuss downstream bioinformatic analyses may provide additional insight into which sequencing platform should be utilized.

## 9. Bioinformatics

DNA sequencers produce “raw” reads which must be subject to computational quality control prior to analysis. During this bioinformatic cleanup process, there exist numerous software options, each designed to produce optimal results for differing scenarios. To guide wildlife investigators with their selection of bioinformatic analyses, this section provides an overview of important steps in 16S gene sequence processing pipelines, and highlights examples of stand-alone and popular all-inclusive methods (See Supplementary Table 4).

First, sequencing adaptors must be removed from raw amplicon reads [using for example, cutadapt (Martin, 2011), trimmomatic (Bolger et al., 2014) or Skewer (Jiang et al., 2014)]. Reads are then quality trimmed [e.g., Cutadapt (Martin, 2011) or TRIMMOMATIC (Bolger et al., 2014)], to filter or truncate error-prone read sequences prior to analysis. Following this, reads are typically subject to paired-end assembly [e.g., PANDAseq (Masella et al., 2012)], which merges mate pairs into longer 16S rRNA gene contigs. Chimeras are then identified and removed from the set of reads [e.g., UCHIME (Edgar et al., 2011), DECIPHER (Wright et al., 2012), or the chimera removal functions in DADA2 (Callahan et al., 2016)].

After these quality filtering steps, sequences can be assigned into operational taxonomic units (OTUs), which are clusters of sequences that are thought to be closely related. This can occur in two general ways: *de novo* (e.g., similarity-based and model-based) and reference-based (e.g., open-reference and closed-reference). Although OTUs can be created in different ways, studies have demonstrated that *de novo* methods, which do not rely on information from a database, outperform reference-based clustering that leverages database-dependent taxonomy binning (Schloss and Westcott, 2011; Westcott and Schloss, 2015; Schloss, 2016). Furthermore, for comparisons between different *de novo* based methods that use sequence similarity to cluster sequences into OTUs, average neighbor clustering – which averages the differences between pairs of sequences – was found to be the most robust method (Schloss and Westcott, 2011; Schloss, 2016). Additionally, when OTU clustering was applied to human twin gut microbiomes, *de novo* clustering identified a higher number of heritable OTUs between twin pairs than other approaches (Jackson et al., 2016), which improved the power of the analysis.

DADA2 (Callahan et al., 2016) and Deblur (Amir et al., 2017) provide an alternative *de novo* clustering approach that does not rely on sequence similarity to assign sequences to OTUs. Rather, these approaches resolve differences between reads that result from sequencing error to resolve the total set distinct biological sequence variants observed in the data. In so doing, these approaches identify specific amplicon sequence variants (ASVs) that preserve fine-scale variation between sequences, which may be lost during sequence similarity based OTU clustering. However, this approach may be subject to sensitivities that obscure detection of singleton OTUs (i.e., those with only one representative sequence in the data set; Callahan et al., 2016). In an analysis incorporating three denoising pipelines (DADA2, Deblur and UNOISE3), Nearing et al. (2018) showed with mock community data, that the number of ASVs produced varied considerably across the pipelines, with DADA2 finding the most ASVs when using real datasets. These discrepancies could have a significant effect on α-diversity metrics. However, the three packages gave consistent per-sample microbial compositions, a result echoed by Glassman and Martiny (2018) who found β-diversity patterns to be robust to the OTU clustering procedure implemented. Despite the differences in ASV count, researchers should also evaluate the financial costs and time constraints associated with the choice of denoising software. DADA2 and Deblur are both open-source and freely available, whereas UNOISE3 is closed-source, but is by far the fastest in terms of analysis run time (1,200 times and 15 times the speed of DADA2 and Deblur, respectively; Nearing et al., 2018).

It is also worth mentioning that UNOISE3 and DADA2 produce ASV output that depends on the input given pool of samples as compared to Deblur, which using a denoising algorithm based on a reference set (Amir et al., 2017). This ensures that all sequences from different samples are denoised independently when considering all other samples in the run. In contrast UNOISE3 and DADA2, denoise based on the current sample pool, and therefore denoise profiles are also a function of the samples that are present (Amir et al., 2017).

Once ASVs or OTU-clustered representative sequences have been produced, they are aligned to enable comparisons between the sequences, assign taxonomy, or construct phylogenetic trees. Three primary algorithms that are commonly used in nucleotide alignments: *de novo* pairwise, *de novo* multiple sequence, and profile-based alignments each offer differing levels of speed and accuracy (Schloss, 2009). Before or after alignment, sequences can be taxonomically annotated using SILVA (Yarza et al., 2008), Greengenes (DeSantis et al., 2006), or Ribosomal Database Project (RDP, Cole et al., 2009) 16S rRNA gene sequence databases. Each 16S database contains sequences with varying levels of alignment quality and phylogenetic diversity (Schloss, 2010) that result in environment-specific taxonomic classification accuracy. For example, SILVA-based taxonomic classification classifies human fecal microbiomes and soil samples with greater accuracy than Greengenes or RDP while RDP-based taxonomic classification better classifies mouse feces (Schloss et al., 2016).

As noted above, it is increasingly appreciated that the nature of microbiome data is compositional (Gloor et al., 2017; Silverman et al., 2017; Weiss et al., 2017) with most studies comparing the relative abundances of taxa (Silverman et al., 2017). Traditional statistical methods assume that the nature of sequencing data is ecological (Gloor et al., 2017), with reads/sample being comparable to biological sampling effort (Weiss et al., 2017; Pannoni et al., 2022). Within one sequencing run, the library size total number of reads per sample can vary by orders of magnitude (McMurdie and Holmes, 2014; Weiss et al., 2017) and often contain many zeros (Weiss et al., 2017). As such, numerous methods to normalize microbiome data have been developed to reduce statistical artifacts produced during analysis and address the compositional nature of the data. Some normalization techniques are mentioned below, but this is not discussed extensively in this review, as this area of microbiome research is constantly evolving and currently there is no consensus as to the best method for library normalization (Pannoni et al., 2022).

Two widely used well-known methods include normalizing using proportions and rarefaction (McKnight et al., 2019). Normalizing using proportions involves dividing the reads in each individual OTU or ASV by the total number of reads in the sample, whereas rarefaction randomly subsamples each sample to the lowest read depth of all samples (McKnight et al., 2019). These methods are seemingly losing favor. For example, rarefaction, leads to the loss of available valid data (McMurdie and Holmes, 2014) and purportedly has a high false discovery rate (Lin and Peddada, 2020). Normalizing using proportions has been criticized as it does not account for heteroskedasticity in the data (Weiss et al., 2017; McKnight et al., 2019). However, rarefaction, compared to other methods based on presence or absence, has been shown to better cluster samples based on biological origin (Weiss et al., 2017). Similarly, McKnight et al. (2019) showed in a study investigating the best normalization methods for microbiome data from an ecological viewpoint, that both normalization of proportions and rarefaction were useful for producing more accurate comparisons among communities, with normalization by proportions found to be the best method overall. Other methods, such as Compositional Data Analysis [CoDA; See Tsilimigras and Fodor (2016) for a review of some CoDA methods], variance stabilization transformation (VST; McMurdie and Holmes, 2014) and more recently, Analysis of Compositions of Microbiomes with Bias Correction (ANCOM-BC; Lin and Peddada, 2020) all have certain analytical advantages and disadvantages (Pannoni et al., 2022). It is important that investigators follow the current literature to determine the potential advantages and pitfalls of newly developed methodologies to ascertain the best solution for their data analysis.

While bioinformaticians can implement these procedures through custom software pipelines to string together these vital informatic processes, there exist several software packages that expedite these steps and bring added uniformity between studies. Of the most commonly used software suites, QIIME (Caporaso et al., 2010) is OTU-based, while DADA2 (Callahan et al., 2016) and most recently QIIME 2 (Bolyen et al., 2019) produce ASVs. Mothur (Schloss et al., 2009) allows the user to choose either an OTU-clustering or ASV approach, depending on preference. A recent review of 6 different pipelines, three OTU-based (QIIME-uClust, mothur & USEARCH-UPARSE) and three ASV-based (DADA2, QIIME2-Deblur & USEARCH-UNOISE3), showed that the ASV-based pipelines had higher specificity (low production of spurious results) than OTU-based pipelines (Prodan et al., 2020). Within the ASV-based pipelines tested, USEARCH-UNOISE3 performed best overall with both high sensitivity (ability to accurately detect true OTUs/ASVs) and good specificity. DADA2 was recommended for studies on closely related strains owing to its high sensitivity and best resolution. Conversely, the QIIME-uclust pipeline was not recommended owing to there being many spurious OTUs and inflated α-diversity values (Prodan et al., 2020). In the end, regardless of the sequencing technology and software selection, inclusion of quality trimming, error correction and read assembly can significantly reduce substitution errors (Schirmer et al., 2015).

The output from these pipelines or software platforms is a matrix relating features (taxa or genes) to the samples (Knight et al., 2018). Generally, microbial community diversity can be measured quantitatively (assessing how relative abundance of taxa is associated with changes in the microbial community) or qualitatively (e.g., presence / absence). We have not delved into higher level analyses (such as α- and β-diversity, PERMANOVA, unweighted and weighted Unifrac) in this review. For a succinct review and more information on these analyses, please consult Knight et al. (2018). As many of these analyses require a phylogenetic tree, we have reviewed phylogenetics and the construction of phylogenetic trees.

## 10. Phylogenetics and ecophylogenetics

Once sequences are processed, filtered, and clustered into OTUs or ASVs, phylogenies can be reconstructed from alignments of representative sequences of each OTU or ASV, providing additional insights into microbial communities. Microbial phylogenetic trees allow for the calculation of evolutionarily informed measures of microbial β-diversity (Lozupone and Knight, 2005), and identification of phylogenetic signal (Gaulke et al., 2018). If considering a diverse set of hosts, combination of microbial and host phylogeny can be used to test for co-phylogenetic signals, as well as for modeling of host traits (Washburne et al., 2017). Microbial phylogenetic trees have been shown to vary based on gene, region, sequence length, alignment, diversity, and reconstruction method. To draw meaningful conclusions from these tools which rely on phylogenies, researchers must be aware of the methodological sources of phylogenetic error that may impact their results.

Phylogenetic reconstruction using different hypervariable regions of the 16S gene will yield differing levels of taxonomic resolution which vary by taxonomic lineage. For example, the 16S rRNA gene is known to be unable to differentiate between species within Bacteroidaceae and Bifidobacteriaceae (Moeller et al., 2016) and no hypervariable region was able to recapture the same set of diversity when compared to the full length 16S rRNA gene (Johnson et al., 2019). Thus, alternative markers should be used when taxa of biological interest are known to have poor separation with 16S gene sequences. Longer sequences are better able to recapitulate full-length genetic variation (Schloss, 2010), increase the proportion of correct trees (Graybeal, 1998), improve branch-length calculations (Rosenberg and Kumar, 2003), and more accurately represent the phylogenetic distance of full-length phylogenies (Ragan-Kelley et al., 2013). However, due to potentially uninformative stretches within genes, analyzing the appropriate region(s) of a gene that yield discriminatory power between taxa has a greater effect on phylogenetic inferences than increasing sequence length (Martin et al., 1995). In addition, and to increase phylogenetic accuracy, it is critical to trim sequences to the same starting and ending regions, as different regions of genes do not mutate at uniform rates (Schloss, 2010). The ability of different 16S hypervariable regions to compute community diversity metrics is discussed in a prior section.

Other limitations of phylogenetic reconstruction using the 16S gene include limitations of using the 16S marker rRNA gene itself. For example, it has been shown that while very rare, it is possible for the 16S gene to be horizontally transmitted between species (Wang and Zhang, 2000; Acinas et al., 2004; Kitahara and Miyazaki, 2013; Tian et al., 2015). There is also evidence to suggest heterotachy (lineage-specific evolutionary rates) within the 16S gene, resulting in complications of phylogenetic interpretation. Despite these limitations, however, the 16S rRNA gene has been used for over 30 years to define phylogenetic relationships of microorganisms (Woese, 1987). In the future longer read technologies may allow for phylogenetic reconstruction using full length sequences or sets of core housekeeping genes shared across many genomes.

There are two main flavors of phylogenetic tree construction: (1) sequence placement approaches onto a phylogenetic reference tree, and (2) de-novo phylogenetic tree construction. Sequence placement approaches effectively use a reference phylogenetic tree to “place” sequences into phylogenetic context with some measure of certainty. Various algorithms exist which utilize different underlying statistical frameworks to map sequences to reference trees such as maximum likelihood [e.g., Evolutionary Placement Algorithm (Berger et al., 2011) and pplacer (Matsen et al., 2010)] or Hidden Markov Models [e.g., SATé-enabled phylogenetic placement (Janssen et al., 2018)]. De-novo phylogenetic tree approaches build a novel tree from sequences using a variety of phylogenetic reconstruction methods to model evolutionary relationships from sequences. The relatively short sequence obtained upon resolving ASV or OTU (e.g., 150 nucleotides) fragments in combination with the fact that the 16S gene is relatively highly conserved across microbes, present the problem that they may not contain sufficient phylogenetic signal to reproduce an accurate phylogenetic tree. Various strategies, such as inclusion of full-length reference sequences, have been shown to allow for more accurate phylogenetic tree construction despite this limitation (O’Dwyer et al., 2015; Gaulke et al., 2018).

There are four primary types of denovo phylogenetic reconstruction methods that model evolutionary relationships from aligned sequences: distance, parsimony, maximum likelihood, and Bayesian inference. Distance-based methods such as neighbor joining (Saitou and Nei, 1987) or minimum-evolution (Rzhetsky and Nei, 1992) rely on a distance matrix composed of all taxa, whereas maximum parsimony methods minimize the number of evolutionary events predicted in the final phylogeny (Felsenstein, 2004). Both maximum likelihood and Bayesian inference employ probability-based statistical approaches to determine the optimal tree. Maximum likelihood methods determine the tree that has the highest probability of depicting evolutionary history based on the likelihood function, while Bayesian inference uses posterior probabilities to optimize topology (Svennblad et al., 2006).

The accuracy of the reconstruction method depends on substitution rate, number of sites, and number of taxa (Rosenberg and Kumar, 2001, 2003). Of note within phylogenetic construction of sequences is that it is essential to ensure that artificial sequences (e.g., adaptors used for sequencing, amplicon primer regions) are removed prior to phylogenetic tree assembly since inclusion can lead to spurious associations between sequences (Arnold et al., 2022; Davis et al., 2022). Generally, maximum likelihood and Bayesian methods reconstruct phylogenies most accurately, followed by maximum parsimony and neighbor-joining, respectively (Rosenberg and Kumar, 2001; Ogden and Rosenberg, 2006; Price et al., 2010). Currently, some of the most popular software used in microbiome studies for phylogenetic tree reconstruction are FastTree2 (Price et al., 2010) RaxML (Stamatakis et al., 2012; Stamatakis, 2014) using maximum likelihood methods, and BEAST (Drummond and Rambaut, 2007) for Bayesian-based tree construction. Recently released RaxML-NG (Kozlov et al., 2019) and IQ-TREE2 (Minh et al., 2020) appear promising as they boast a number of improvements including the accuracy of maximum likelihood with greatly reduced computational time compared to prior options.

While different methods of phylogenetic tree reconstruction will provide varying levels of accuracy, phylogenies in general are highly dependent on the quality of sequence alignment. Morrison and Ellis (1997) found that sequence alignments accounted for more phylogenetic variation than choice of tree-building method. Schloss (2009, 2010) has conducted extensive studies that demonstrate differences in alignment quality between full-length 16S databases that are commonly used for reference-based alignment and found that poor quality alignments inflate phylogenetic diversity. As a result, the lower quality variable region alignments in the Greengenes database predict higher genetic diversity, richness, and phylogenetic diversity than alignments using the SILVA and RDP databases (Schloss, 2010). Errors in topology from poor alignments also become magnified in phylogenies with shallow diversity (Ogden and Rosenberg, 2006) and both sequence diversity and the number of lineages have been shown to impact phylogenetic accuracy [reviewed in Hillis et al., 2003 and Nabhan and Sarkar, 2012].

With the exception of common community level β-diversity metrics (e.g., unifrac), typical microbial analyses remain largely phylogenetically unaware. A consequence of phylogenetic-agnostic approaches is that meaningful patterns between microbial communities and ecological covariates are lost. Ecophylogenetics is a burgeoning field seeking a unified analytical framework of microbial evolutionary history (i.e., phylogeny) and ecological community patterns. In combination, ecophylogenetics is able to link evolutionary related groups of microbes to ecosystem services of interest (Mouquet et al., 2012; Gaulke et al., 2018). Ecology and evolution are inherently linked with one another; evolution results in diversification of monophyletic microbial lineages, or clades, within a community which interact with ecological ecosystem parameters. In turn, ecological parameters create selection of microbial lineages, influencing microbial community composition and providing opportunity for microbial functional specialization and speciation events.

Vast opportunity exists to apply microbial ecophylogenetic methods within a wildlife and disease ecology setting to (1) determine how microbial clades are selected for based on host (e.g., host immune status, parasitic burden) and environmental parameters (e.g., population fragmentation, anthropogenic factors) and (2) understand how radiation of microbial clades within a host impacts community assembly and host fitness (e.g., host energy balance, disease susceptibility; Prosser et al., 2007). Monophyletic clades, clades which contain descendants all from a common ancestor, that are highly prevalent across individuals represent lineages which may hold conserved traits key to microbial actions on host physiology. Conserved microbial traits within the lineage may also facilitate the clade’s distribution across hosts. For example, identification of a microbial clade strongly associated with host fitness provides novel hypotheses about conserved microbial traits which influence host success within a particular environment. Conserved clades may be important candidate lineages to pinpoint for conservation management monitoring and preservation strategies.

The ClaaTU workflow is an open-source tool that has been developed to aid microbiome researchers in ecophylogenetic analysis to identify Cladal Taxonomic Units, which collectively manifest an association with ecological parameters of interest (Gaulke et al., 2018; Couch et al., 2020; Sharpton et al., 2021). ClaaTU is a brute-force algorithm that conducts a root-to-tip traversal of a phylogenetic tree assembled from microbial sequences derived from a set of microbial communities (e.g., the OTU output table from DADA2). ClaaTU considers every lineage within a phylogeny to identify the ecological distribution of monophyletic groups of taxa within the samples of interest. Finally, a phylogenetically informed permutation test determines if a given clade is more prevalent than expected by chance across a set of samples, indicating ecological conservation.

Overall, maximizing the accuracy of phylogenetic analyses is complex and requires researchers to understand how each decision in their analyses may affect potential conclusions. Generally, to improve phylogenetic accuracy the most important considerations are the gene region of interest and the alignment algorithm. Secondarily, tree reconstruction method, sequence length, number of lineages, and diversity between lineages influence phylogenetic accuracy. Additional considerations must be made if conducting clade-based analyses due to their dependence on rooted phylogenies.

## 11. Conclusion

The incorporation of the 16S rRNA gene into the analytical repertoire of wildlife investigators has provided powerful, inexpensive insights into gut microbial communities and expanded our understanding of their role in wildlife ecology, health and potentially even population dynamics. However, the procurement of samples in the field can be costly, often including travel to remote sensitive areas or the capture and handling of animals (Cattet et al., 2008), in some cases threatened species. Thus, it is imperative that wildlife veterinarians and researchers wishing to embark on a study that includes 16S rRNA gene analyses have a thorough understanding of the numerous sources of error that can compromise studies, the various options available to avoid these errors, and how different choices affect research outcomes. While studies are calling for a standardized protocol to aid comparisons across microbiome research (Greathouse et al., 2019), to the best of our knowledge, there is currently no universal consensus regarding the best methodological approach for microbiome analyses, possibly due to the fact that different studies manifest different goals and constraints. We can regardless look at the research summarized here to zero-in on major points for consideration and derive recommendations of practice.

For a wildlife researcher, perhaps the most critical element in a microbiome study is the use of as-sterile-as-possible sample collection techniques in the field, thereby reducing the risk of cross-contamination of samples. Extracting DNA from fresh fecal samples circumvents potential storage and preservation effects, although in the event of delayed sample processing, cold storage or cryopreservation at −80°C without a buffer is considered the gold standard at reducing potential changes to the microbial community composition (Vandeputte et al., 2017; Carruthers et al., 2019; Moossavi et al., 2019; Marotz et al., 2021). In cases where freezing may not be an option, such as if samples need to be transported internationally where preservation methods require buffer solutions, it is important to know the limitations of supplies and sample storage conditions. Many of the buffer solutions have only been tested in temperature-controlled laboratories and may fare differently in more extreme environments. Furthermore, choice of buffer solution should consider the long-term storage of samples should transport between sample collection site and storage destination be delayed, such as could happen if samples are delayed at customs or during shipping. These recommended sample collection and storage methods do not inherently consider their potential effect on other uses of the samples. For example, to study the transcriptome or metabolome it is critical to either snap freeze or preserve samples in a suitable buffer that maintains the integrity of the RNA (Camacho-Sanchez et al., 2013). Thus, when designing a gut microbiome study, and sample collection and storage protocols, the potential future uses for samples should be considered.

When extracting DNA from samples, the use of kit-based DNA extraction methods may be preferable to researchers new to the field, as owing to their consistency of approach, they can reduce variability and improve cross-study comparisons. To account for potential sources of experimental contamination, we suggest the inclusion of negative controls with all sample sets that are ultimately subjected to DNA sequencing and analysis, especially when processing low biomass communities. Moreover, the use of mock communities can serve as a strong quality control to identify error-driven outliers within samples (Bender et al., 2018) and to quantify kit or batch effects. We also stress that mechanical lysis should be integrated to ensure that maximum diversity within the community is captured. Following DNA extraction, optimal primer selection may be microbial community specific, but our review of current best practice suggests that reads that include portions of the V4 hypervariable region appear to frequently provide improved discriminatory power.

Finally, during bioinformatic processing, we suggest careful attention be paid during the various pre-processing steps (see Figure 1). While excellent bioinformatic pipelines exist to help streamline bioinformatic analyses of these data, we recommend that researchers new to the field collaborate with bioinformaticians that can help ensure that these pipelines are appropriately applied to their data of interest. It would also be prudent for researchers to work through these pipelines using standardized data sets, such as those in the Earth Microbiome Project (Caporaso et al., 2018) or the Microbiome Quality Control Protocols (Sinha et al., 2017), to assist with understanding the techniques and interpretation of the results (Knight et al., 2018). Should researchers wish to embark on a meta-analysis, it is imperative that they are cognizant of and analytically correct for study-effects that may diminish cross-study comparisons (Armour et al., 2019). Ultimately, we stress that methodological consistency between samples within a study is of paramount importance to reduce sample-specific effects.

In this paper, we have outlined several broad recommendations and key considerations to assist wildlife researchers in designing suitable gut microbiome studies. Although this is not a definitive guide, owing to the constant improvement of techniques and software available, we hope that this paper will prove a useful resource to wildlife researchers hoping to incorporate microbiome analyses into their research design.

## Author contributions

LC: conceptualization, writing and manuscript preparation, and review and editing. IH: conceptualization, writing, original draft manuscript and review and editing. QW, HA, and KS: writing, editing, and revision. KK: editing and revision. AJ and BB: editing, revision, and supervision. TS: conceptualization, writing, editing, revision, and supervision. All authors contributed to the article and approved the submitted version.

## Funding

This research was supported by the Morris Animal Foundation (Postdoc Fellowship grant D18ZO-405), the National Science Foundation (grant DEB 1557192) and by an NSF-NIH-NIFA Ecology and Evolution of Infectious Disease grant number DEB 1911994 and by the UK Biotechnology and Biological Sciences Research Council as grant number BB/T011416/1.

## Conflict of interest

The authors declare that the research was conducted in the absence of any commercial or financial relationships that could be construed as a potential conflict of interest.

## Publisher’s note

All claims expressed in this article are solely those of the authors and do not necessarily represent those of their affiliated organizations, or those of the publisher, the editors and the reviewers. Any product that may be evaluated in this article, or claim that may be made by its manufacturer, is not guaranteed or endorsed by the publisher.

## Footnotes

^1^ https://earthmicrobiome.org/protocols-and-standards/16s/
