# Supplementary material for: Best practice for wildlife gut microbiome research: A comprehensive review of methodology for 16S rRNA gene investigations
Source: Front Microbiol. 2023 Feb 22;14:1092216. doi: 10.3389/fmicb.2023.1092216 (PMC9992432; doi:10.3389/fmicb.2023.1092216)
Supplement: Supplementary file 2 [file Data_Sheet_1.pdf]

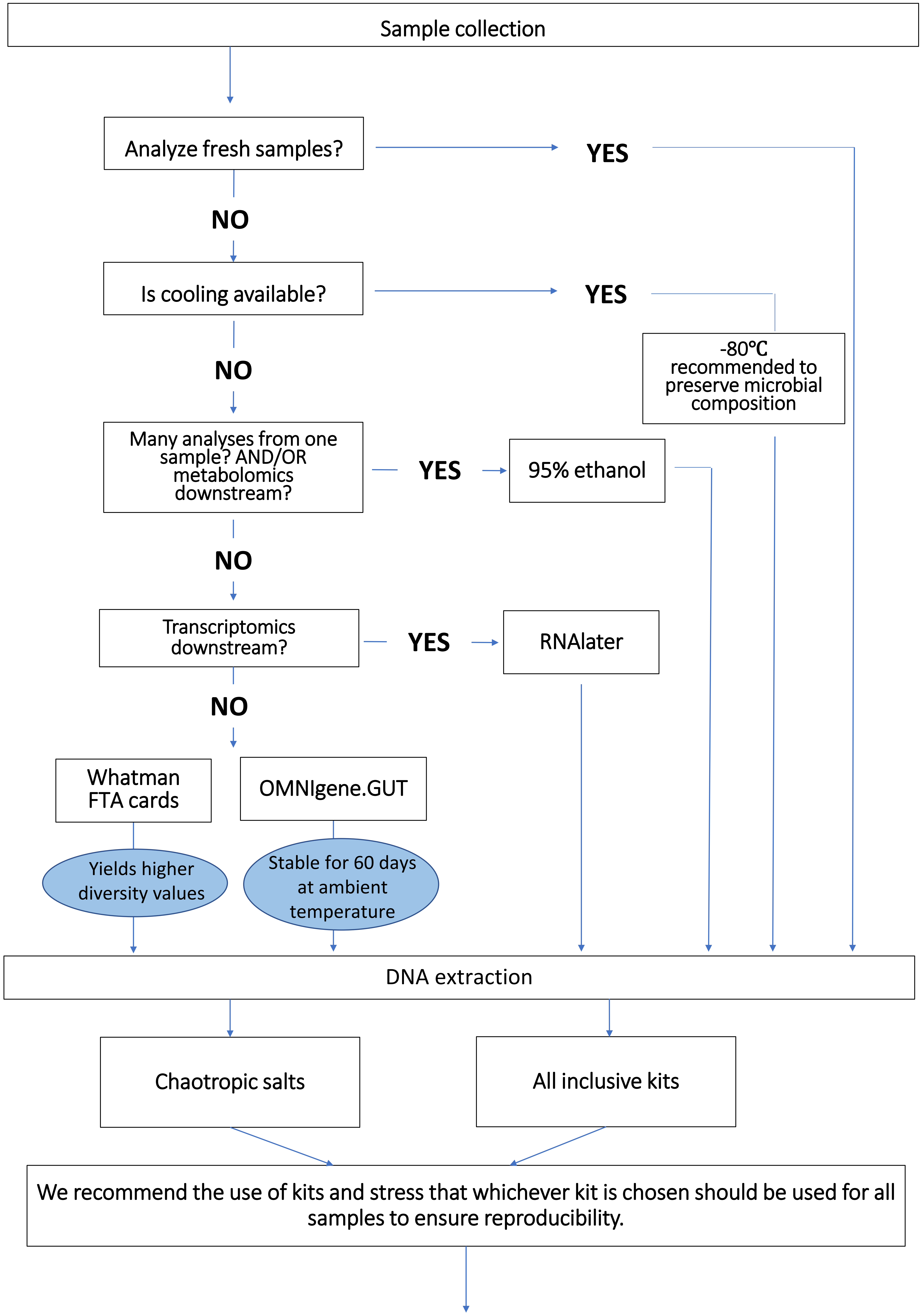
Sample collection
Analyze fresh samples?
YES
NO
Is cooling available?
YES
-80℃ recommended to preserve microbial composition
NO
Many analyses from one sample? AND/OR metabolomics downstream?
YES
95% ethanol
NO
Transcriptomics downstream?
YES
RNAlater
NO
Whatman FTA cards
OMNIgene.GUT
Yields higher diversity values
Stable for 60 days at ambient temperature
DNA extraction
Chaotropic salts
All inclusive kits
We recommend the use of kits and stress that whichever kit is chosen should be used for all samples to ensure reproducibility.

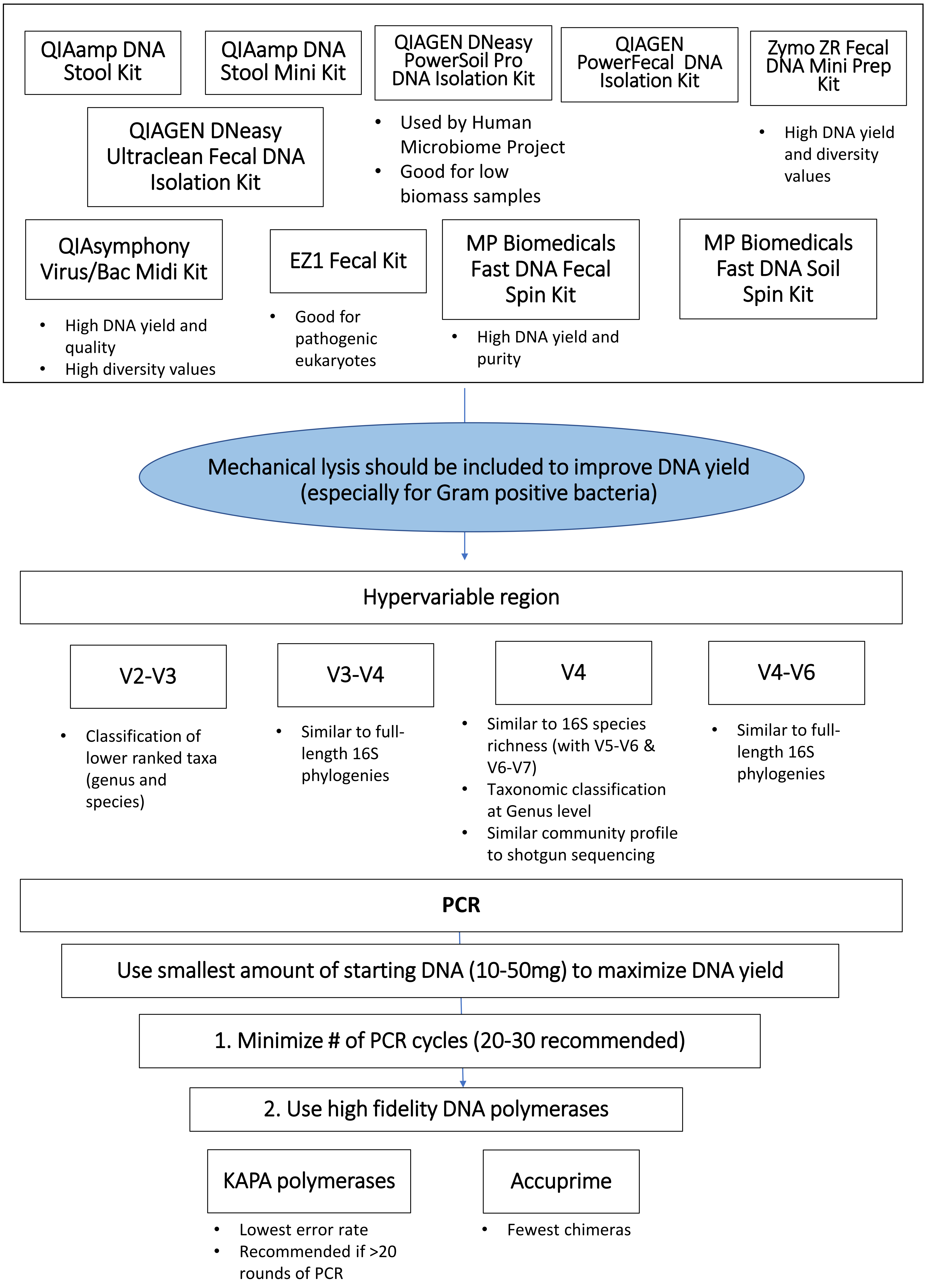
QIAamp DNA Stool Kit
QIAamp DNA Stool Mini Kit
QIAGEN DNeasy PowerSoil Pro DNA Isolation Kit
• Used by Human Microbiome Project
• Good for low biomass samples
QIAGEN PowerFecal DNA Isolation Kit
Zymo ZR Fecal DNA Mini Prep Kit
• High DNA yield and diversity values
QIAGEN DNeasy Ultraclean Fecal DNA Isolation Kit
QIAsymphony Virus/Bac Midi Kit
• High DNA yield and quality
• High diversity values
EZ1 Fecal Kit
• Good for pathogenic eukaryotes
MP Biomedicals Fast DNA Fecal Spin Kit
• High DNA yield and purity
MP Biomedicals Fast DNA Soil Spin Kit
Mechanical lysis should be included to improve DNA yield (especially for Gram positive bacteria)
Hypervariable region
V2-V3
• Classification of lower ranked taxa (genus and species)
V3-V4
• Similar to full-length 16S phylogenies
V4
• Similar to 16S species richness (with V5-V6 & V6-V7)
• Taxonomic classification at Genus level
• Similar community profile to shotgun sequencing
V4-V6
• Similar to full-length 16S phylogenies
PCR
Use smallest amount of starting DNA (10-50mg) to maximize DNA yield
1. Minimize # of PCR cycles (20-30 recommended)
2. Use high fidelity DNA polymerases
KAPA polymerases
• Lowest error rate
• Recommended if >20 rounds of PCR
Accuprime
• Fewest chimeras

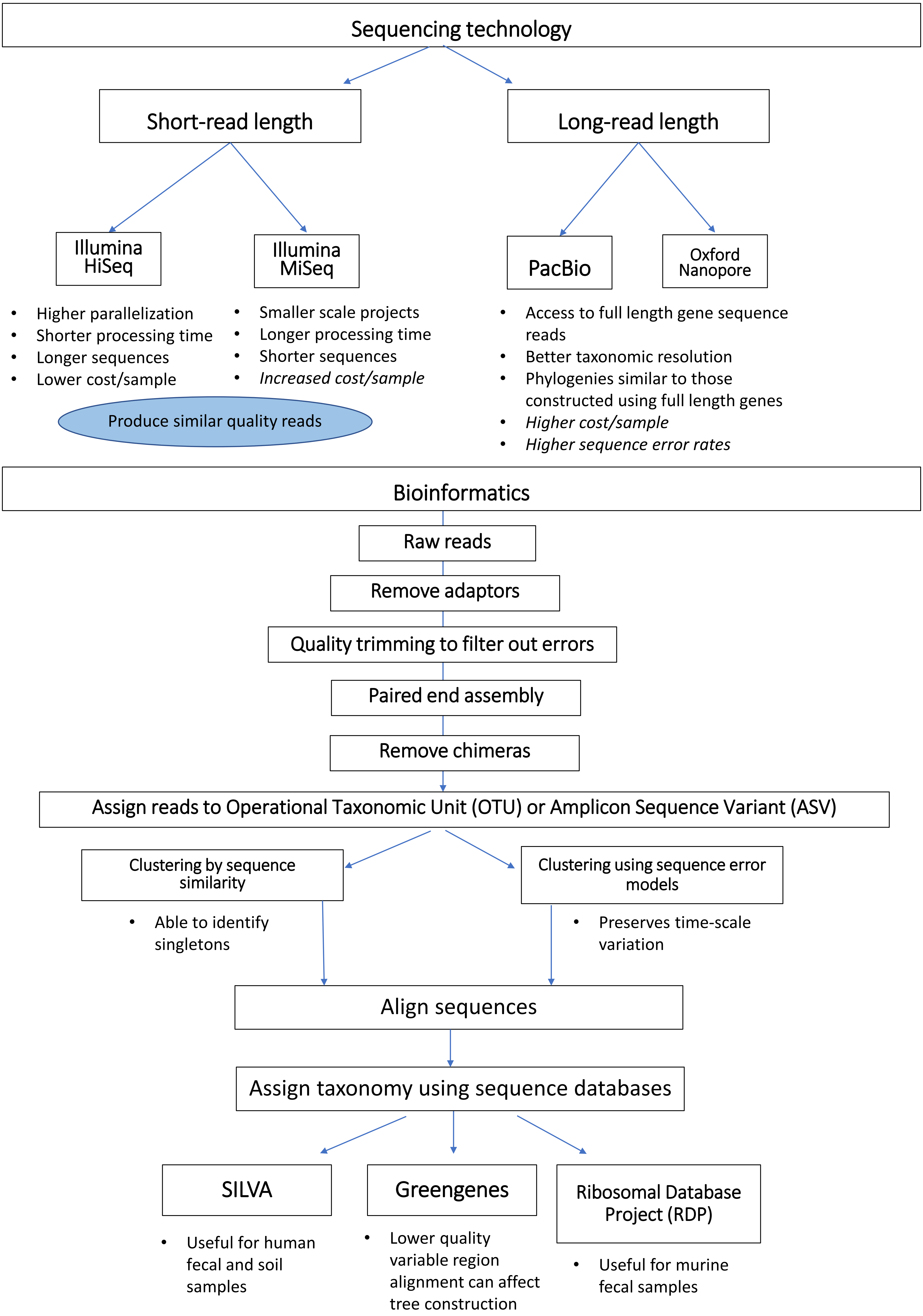
Sequencing technology
Short-read length
Long-read length
Illumina HiSeq
Illumina MiSeq
PacBio
Oxford Nanopore
• Higher parallelization
• Shorter processing time
• Longer sequences
• Lower cost/sample
Produce similar quality reads
• Smaller scale projects
• Longer processing time
• Shorter sequences
• Increased cost/sample
• Access to full length gene sequence reads
• Better taxonomic resolution
• Phylogenies similar to those constructed using full length genes
• Higher cost/sample
• Higher sequence error rates
Bioinformatics
Raw reads
Remove adaptors
Quality trimming to filter out errors
Paired end assembly
Remove chimeras
Assign reads to Operational Taxonomic Unit (OTU) or Amplicon Sequence Variant (ASV)
Clustering by sequence similarity
• Able to identify singletons
Clustering using sequence error models
• Preserves time-scale variation
Align sequences
Assign taxonomy using sequence databases
SILVA
• Useful for human fecal and soil samples
Greengenes
• Lower quality variable region alignment can affect tree construction
Ribosomal Database Project (RDP)
• Useful for murine fecal samples

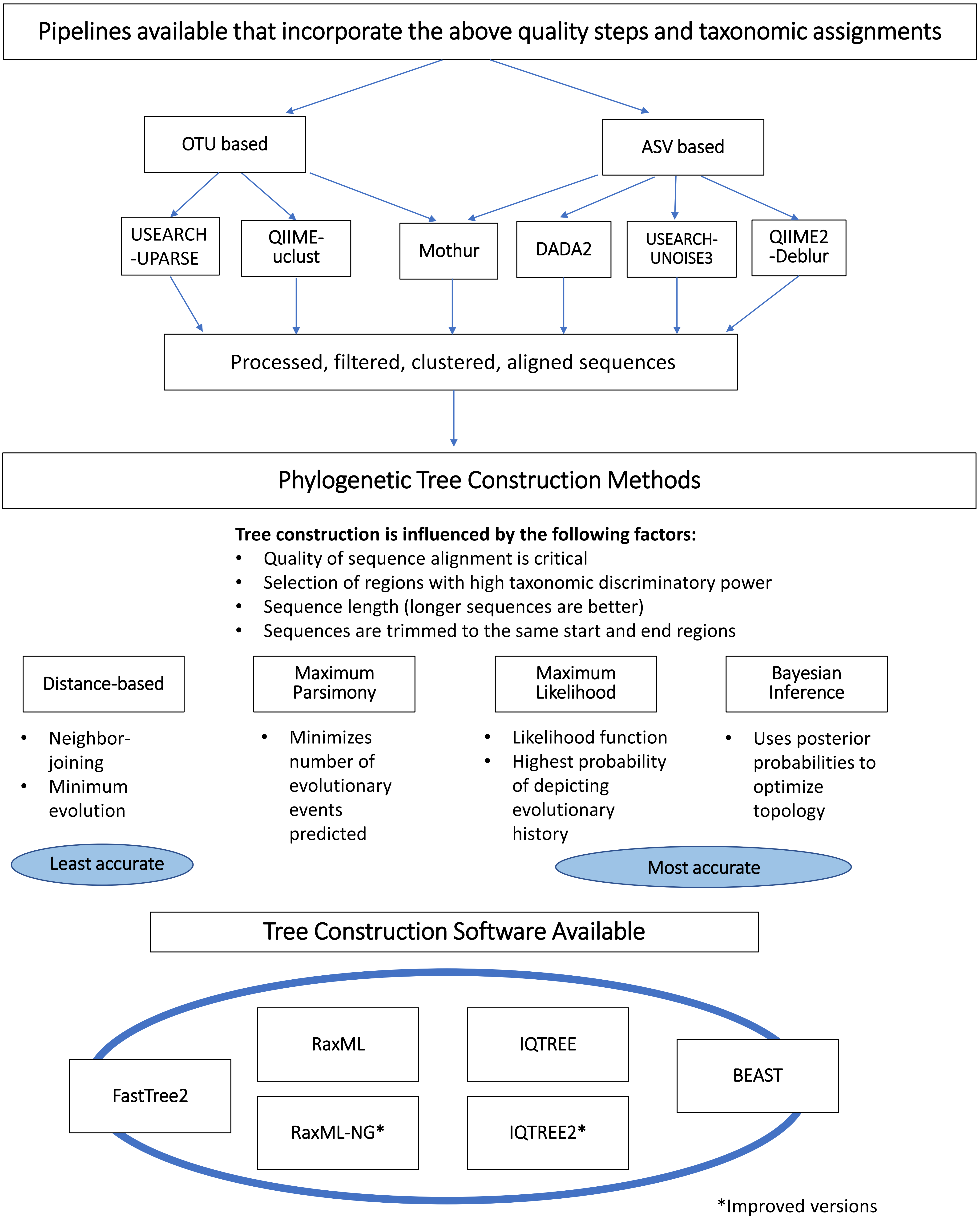

Pipelines available that incorporate the above quality steps and taxonomic assignments
OTU based
ASV based
USEARCH -UPARSE
QIIME-uclust
Mothur
DADA2
USEARCH-UNOISE3
QIIME2 -Deblur
Processed, filtered, clustered, aligned sequences
Phylogenetic Tree Construction Methods
Tree construction is influenced by the following factors:
• Quality of sequence alignment is critical
• Selection of regions with high taxonomic discriminatory power
• Sequence length (longer sequences are better)
• Sequences are trimmed to the same start and end regions
Distance-based
• Neighbor-joining
• Minimum evolution
Maximum Parsimony
• Minimizes number of evolutionary events predicted
Maximum Likelihood
• Likelihood function
• Highest probability of depicting evolutionary history
Bayesian Inference
• Uses posterior probabilities to optimize topology
Least accurate
Most accurate
Tree Construction Software Available
FastTree2
RaxML
RaxML-NG*
IQTREE
IQTREE2*
BEAST
*Improved versions
